# Supplementary material for: The Systems Biology Research Tool: evolvable open-source software
Source: BMC Syst Biol. 2008 Jun 29;2:55. doi: 10.1186/1752-0509-2-55 (PMC2446383; doi:10.1186/1752-0509-2-55)
Supplement: Additional file 1 — SBRT Archive. An archive of the current version of the Systems Biology Research Tool. [file 1752-0509-2-55-S1.zip › sbrt-1.4.0/doc/developers_guide/api/sbrt/kernel/fba/proc/RxnDeletionProcess.html]

RxnDeletionProcess


|  |  |  |
| --- | --- | --- |
| **Overview** \| **Package** \| **Class** \| **Use** \| **Tree** \| **Deprecated** \| **Index** \| **Help** | |  |
| **PREV CLASS**   **NEXT CLASS** | **FRAMES**    **NO FRAMES**     **All Classes** |  |
| SUMMARY: NESTED \| FIELD \| CONSTR \| METHOD | DETAIL: FIELD \| CONSTR \| METHOD |  |


---


## sbrt.kernel.fba.proc Class RxnDeletionProcess

```
java.lang.Object
  sbrt.kernel.fba.proc.FbaProcess<FbaOptComputer.Results>
      sbrt.kernel.fba.proc.RxnDeletionProcess
```

**All Implemented Interfaces:**: KernelProcess<FbaOptComputer.Results>, MonitoredProcess<FbaOptComputer.Results>

---

``` public final class RxnDeletionProcess extends FbaProcess<FbaOptComputer.Results> implements MonitoredProcess<FbaOptComputer.Results> ```

This class is used to perform reaction deletion experiments.

**Author:**
:   This class was written and documented by Jeremiah Wright
    while in the Wagner lab.

---

**Constructor Summary**

`RxnDeletionProcess(RxnDeletionComputer computer)`
  
          Constructs a new reaction deletion experiment.


| **Method Summary** | |
| --- | --- |
| `Fluxome` | `getFluxome()`             Returns the fluxome used by the reaction deletion computer. |
| `MathExpr<java.lang.String>` | `getObjectiveFunction()`             Returns the current objective function. |
| `OptimizationSense` | `getOptimizationSense()`             Returns the current optimization sense. |
| `ProgressMonitor` | `getProgress()`             Returns a progress monitor for this experiment. |
| `double` | `getZeroCutoff()`             Returns the current zero cutoff used by the reaction deletion computer. |
| `void` | `run(ResultsManager<FbaOptComputer.Results> resultsManager)`             Runs this process and adds the generated results to the provided results manager via its `ResultsManager.addData(Object)` method. |
| `void` | `setObjectiveFunction(MathExpr<java.lang.String> objective)`             Sets the objective function to the provided mathematical expression. |
| `void` | `setOptimizationSense(OptimizationSense sense)`             Sets the optimization sense to the provided value. |
| `void` | `setRxnDeletionSource(java.lang.Iterable<java.util.Set<java.lang.String>> deletionSource)`             Sets the reaction deletion input source. |
| `void` | `setZeroCutoff(double zeroCutoff)`             Sets the zero cutoff to the provided value. |

**Methods inherited from class sbrt.kernel.fba.proc.FbaProcess**

`toString`

**Methods inherited from class java.lang.Object**

`clone, equals, finalize, getClass, hashCode, notify, notifyAll, wait, wait, wait`

**Constructor Detail**

### RxnDeletionProcess

```
public RxnDeletionProcess(RxnDeletionComputer computer)
```

:   Constructs a new reaction deletion experiment.

    **Parameters:**: `computer` - the computer with which all results will be computed.


**Method Detail**

### getFluxome

```
public Fluxome getFluxome()
```

:   Returns the fluxome used by the reaction deletion computer.

    :   **Specified by:**: `getFluxome` in class `FbaProcess<FbaOptComputer.Results>`
    :   **Returns:**: the fluxome used by the reaction deletion computer.

---


### getZeroCutoff

```
public double getZeroCutoff()
```

:   Returns the current zero cutoff used by the reaction deletion computer.

    :   **Returns:**: the current zero cutoff used by the reaction deletion computer.

---


### getObjectiveFunction

```
public MathExpr<java.lang.String> getObjectiveFunction()
```

:   Returns the current objective function.

    :   **Returns:**: the current objective function.

---


### getOptimizationSense

```
public OptimizationSense getOptimizationSense()
```

:   Returns the current optimization sense.

    :   **Returns:**: the current optimization sense.

---


### setObjectiveFunction

```
public void setObjectiveFunction(MathExpr<java.lang.String> objective)
```

:   Sets the objective function to the provided mathematical expression.

    :   **Parameters:**: `objective` - a mathematical expression of reaction names.

---


### setOptimizationSense

```
public void setOptimizationSense(OptimizationSense sense)
```

:   Sets the optimization sense to the provided value.

    :   **Parameters:**: `sense` - an optimization sense.

---


### setRxnDeletionSource

```
public void setRxnDeletionSource(java.lang.Iterable<java.util.Set<java.lang.String>> deletionSource)
```

:   Sets the reaction deletion input source.

    :   **Parameters:**: `deletionSource` - a source of reaction names sets whose effect upon deletion is to be computed.

---


### setZeroCutoff

```
public void setZeroCutoff(double zeroCutoff)
```

:   Sets the zero cutoff to the provided value. The zero cutoff is the
    value used to decide which computed values are close enough to zero
    to be considered equal to zero. This value is an upper bound and
    should itself be close to zero, like 1E-6 for example.

    :   **Parameters:**: `zeroCutoff` - the zero cutoff.

---


### getProgress

```
public ProgressMonitor getProgress()
```

:   Returns a progress monitor for this experiment.

    :   **Specified by:**: `getProgress` in interface `MonitoredProcess<FbaOptComputer.Results>`
    :   **Returns:**: a progress monitor for this experiment.

---


### run

```
public void run(ResultsManager<FbaOptComputer.Results> resultsManager)
```

:   Runs this process and adds the generated
    results to the provided results manager via
    its `ResultsManager.addData(Object)`
    method.

    A set of reaction names is retrieved from the deletion source, their
    effect upon deletion is computed, and the results are added to the
    provided results manager. This is repeated until the end of the deletion
    source is reached.

    :   **Specified by:**: `run` in interface `KernelProcess<FbaOptComputer.Results>`
    :   **Parameters:**: `resultsManager` - the results manager to which the results will be added.


---


|  |  |  |
| --- | --- | --- |
| **Overview** \| **Package** \| **Class** \| **Use** \| **Tree** \| **Deprecated** \| **Index** \| **Help** | |  |
| **PREV CLASS**   **NEXT CLASS** | **FRAMES**    **NO FRAMES**     **All Classes** |  |
| SUMMARY: NESTED \| FIELD \| CONSTR \| METHOD | DETAIL: FIELD \| CONSTR \| METHOD |  |


---
